# Lateralized power spectra of the EEG as an index of visuospatial attention

**DOI:** 10.2478/v10053-008-0144-7

**Published:** 2013-12-31

**Authors:** Rob H. J. Van der Lubbe, Christian Utzerath

**Affiliations:** 1 Cognitive Psychology and Ergonomics, University of Twente, The Netherlands; 2 Department of Cognitive Psychology, University of Finance and Management in Warsaw, Poland

**Keywords:** visuospatial attention, inhibition, ERPs, ERLs, wavelets

## Abstract

The electroencephalogram (EEG) was measured in an endogenous orienting paradigm where symbolic cues indicated the likely side of to-be-discriminated targets. Combined results of event-related lateralizations (ERLs) and a newly derived measure from wavelet analyses that we applied on the raw EEG and individual event-related potentials (ERPs), the lateralized power spectra (LPS) and the LPS-ERP, respectively, confirmed the common view that endogenous orienting operates by anterior processes, probably originating from the frontal eye fields, modulating processing in parietal and occipital areas. The LPS data indicated that modulation takes place by increased inhibition of the irrelevant visual field and/or disinhibition of the relevant to-be-attended visual field. Combined use of ERLs, the LPS, and the LPS-ERP indicated that most of the involved processes can be characterized as externally evoked, either or not with clear individual differences as some evoked effects were only visible in the LPS-ERERP, whereas few processes seemed to have an internally induced nature. Use of the LPS and the LPS-ERP may be advantageous as it enables to determine the involvement of internally generated lateralized processes that are not strictly bound to an event like stimulus onset.

## INTRODUCTION

Several studies in the past revealed that when either the left or the right hemisphere is likely involved with carrying out a specific process (e.g., attentional orienting, memorization, or the preparation of eye, hand, or leg movements), activity can be extracted from the EEG (electroencephalogram) that is specific to the relevant side: so-called *event-related lateralizations* (ERLs; Wascher & Wauschkuhn, 1996; see also De Kleine & Van der Lubbe, 2011; Van der Lubbe et al., 2000; Van der Lubbe, Neggers, Verleger, & Kenemans, 2006). ERLs are difference waves that can be derived from event-related potentials (ERPs) by employing a double subtraction technique. The computation of ERPs and ERLs assumes that the signal of interest is constant and bound to a specific event (e.g., stimulus onset or emission of a response). Furthermore, it is assumed that noise is random and automatically cancels out by averaging across a sufficient number of trials. Not surprisingly, these assumptions have been questioned as the onset of a process like attentional orienting likely varies over trials (and between individuals), and this variation may be more interesting than pure event-related activity (e.g., see Buzsáki, 2006).^1^ In the current paper, an alternative method was examined by determining lateralized activity based on wavelet analyses of the raw EEG, which we denoted as the lateralized power spectra (LPS). We explored this in a standard paradigm that has often been employed to study attentional orienting and we compared the ERL results with the new LPS findings. Furthermore, the LPS was also determined for the individually obtained ERPs, denoted as the LPS-ERP, which may provide important information regarding the evoked (bound to an external event) or more induced nature (internally generated) of observed lateralized activity. The latter analyses could possibly also reveal evoked effects that are not visible in the ERLs due to individual differences.

Since the 1980’s, the standard way to examine spatial attention and its effects on stimulus processing is the Posner (1980) cuing paradigm. In this paradigm, often a lateral to-be-detected or to-be-discriminated target is preceded by a cue that provides information about the target location. In the case of endogenous orienting (i.e., attention is directed voluntarily), this may concern a centrally displayed cue (often an arrow-like symbol) that predicts the likely locus of the target. Commonly, attended target stimuli are better perceived and receive faster responses than unattended target stimuli (e.g., see Hawkins et al., 1990; Van der Lubbe et al., 2006). However, the precise nature of the attentional mechanism underlying this behavioral benefit is not that well understood.

The currently dominant view is that attention induces sensory gain, referring to the idea that attention boosts target-related neural activity. Indeed, the increased target-induced P1 component on validly as compared to invalidly cued trials is commonly interpreted in terms of sensory gain (e.g., Mishra, Martínez, Schroeder, & Hillyard, 2012; but see Klimesch, 2011). Additionally, it has been argued that attention reduces external noise (by inhibiting distracting information; see Kastner & Ungerleider, 2001), but some authors pointed to the possibility that attention reduces internally generated neural noise (Lu & Dosher, 1998). The latter option is not often highlighted. For example, fMRI data indicate that there may already be changes in V1 while awaiting a to-be attended target (Kastner, Pinsk, De Weerd, Desimone, & Ungerleider, 1999), but these results are generally interpreted as reflecting sensory gain. Furthermore, they have been related to increased neuronal firing rates as observed in single-cell recording studies in monkey extrastriate cortex (see Luck, Chelazzi, Hillyard, & Desimone, 1997). In contrast with this idea of sensory gain, an impressive series of recent EEG studies suggest that inhibition of neural activity may be the crucial mechanism to regulate the ongoing flow of visual information processing (e.g., see Gould, Rushworth, & Nobre, 2011; Rihs, Michel, & Thut, 2009; Sauseng et al., 2005; Thut, Nietzel, Brandt, & Pascual-Leone, 2006; Worden, Foxe, Wang, & Simpson, 2000).

Worden et al. (2000) employed an endogenous orienting paradigm with visual targets and demonstrated that the power of oscillatory activity in the α-band (~8-12 Hz; discovered by Berger, 1929), which has been related to synchronized thalamo-cortical interactions (Lopes da Silva, 1991), crucially depends on the cued side. Specifically, when cues indicated that attention had to be directed to the left or the right visual field (LVF or RVF), increased power was observed above ipsilateral occipital areas shortly before visual target onset, whereas decreased power was observed above contralateral occipital areas. This increased ipsilateral and decreased contralateral power was interpreted as reflecting inhibition of the to-be-ignored visual field and/or the release of inhibition (i.e., disinhibition) of the to-be-attended field, respectively. Thut et al. (2006) demonstrated that this effect occurs irrespective of cue modality by comparing the effects of auditory and visual symbolic cues in a visual detection task. Interestingly, they proposed to use a lateralization index in which the difference in power between ipsilateral and contralateral sites was calculated.

(1) $$\mathrm{lateralization}\mathrm{index}{\left(\alpha \right)}_{t}=\frac{\alpha_{t}\left(\mathrm{PO8}\right)–\alpha_{t}\left(\mathrm{PO7}\right)}{\alpha_{t}\left(\mathrm{PO7}\right)+\alpha_{t}\left(\mathrm{PO8}\right)}$$

In this calculation, the power at time point *t* (α_t_) is determined for two symmetrical electrodes (or a group of symmetrical electrodes) on the scalp above the left and right hemisphere (e.g., PO7 and PO8 above the left and right visual cortex, respectively). Subsequently, the power difference between these electrodes is computed, being scaled by the sum of their powers. This results in a lateralization index of which values range from -1 to +1. A negative value of this index at time point *t* indicates that power at this specific moment is larger above the left electrode (PO7; related to processing of the RVF) than above the right electrode (PO8; related to the LVF), whereas a positive value implies that power is larger above the right than the left electrode. Inthe study of Thut et al. (2006), this index was clearly negative in the cue-target interval shortly before target onset when the left side was cued. This finding seems to reflect inhibition of the RVF as compared to the LVF and/or disinhibition of the LVF relative to the RVF. However, no opposite effect was found when the right side was cued. Although absence of the latter effect (or presence of the effect when the left side was cued) could be due to a general attentional bias or hemispherical differences (discussed below), an alternative possibility is that the use of a detection task with right hand responses may have caused problems. Specifically, as the required response hand in this task is already known, there might be (motor and/or attentional) activity related to this hand during the cue-target interval, which might somehow interfere.^2^ In more recent studies, Rihs et al. (2009) and Gould et al. (2011) used discrimination tasks, but participants were responding only with their right hand, which might still cause problems as attention may have been directed at this side. This potential confound can easily be avoided by using a discrimination task requiring left and right hand responses. Another issue concerns the possibility that general hemispherical differences (e.g., see Verleger, migasiewicz, & Möller, 2011) complicate the lateralization index. For example, the right hemisphere is thought to be actively involved (i.e., disinhibited) when attention has to be directed towards any location, which may increase hemispherical differences in the case of left cues and reduce these differences in the case of right cues. This issue can be solved by using a double subtraction rather than a single subtraction, like has been done to compute the lateralized readiness potential (LRP; see e.g., Coles, 1989; De Jong, Wierda, Mulder, & Mulder, 1988; Eimer, 1998; Gratton, Coles, Sirevaag, Eriksen, & Donchin, 1988).

Our interest was directed at the further development of a measure sensitive to spatial attention during the whole cue-target interval along the lines suggested by Thut et al. (2006). Earlier EEG studies did not focus on specific spectra but examined event-related lateralizations of ERPs, the aforementioned ERLs. In the study of Harter, Miller, Price, LaLonde, and Keyes (1989) and in other studies (e.g., Eimer, 1995; Hopf & Mangun, 2000; Nobre, Sebestyen, & Miniussi, 2000; Van der Lubbe et al., 2006; Yamaguchi, Tsuchiya, & Kobayshi, 1994, 1995), seve-ral ERL components were distinguished, which were thought to be related to specific cognitive processes.^3^ In the study of Van der Lubbe et al. (2006), ERLs were computed on the basis of ERPs in a similar way as the LRP (see Wascher & Wauschkuhn, 1996), by extending this method to all symmetrical electrodes above the left and right hemispheres.

(2) $${\mathrm{ERL}}_{t}=\frac{\left(\text{left cues}\left(V_{t}\left(\mathrm{PO8}\right)–V_{t}\left(\mathrm{PO7}\right)\right)\right)+\left(\text{right cues}\left(V_{t}\left(\mathrm{PO7}\right)–\left(\mathrm{PO8}\right)\right)\right)}{2}$$

In this calculation, for left cues, the voltage *V* at time point *t* after cue onset at the electrode ipsilateral (PO7) to the to-be attended side is subtracted from the voltage at time point *t* at the electrode contralateral (PO8) to the to-be attended side, and the same procedure is followed for right cues. Subsequently, these values are averaged. By applyingthis method to all available lateral electrodes, a contra-ipsilateral topographic map can be determined. After an additional inversion a mirror-symmetric map can be constructed for a specific time window (see Van der Lubbe et al., 2006). An important feature of this method is that all neuronal activity unrelated to the focus of attention is cancelled out, making this index highly specific for changes in spatial attention. Application of this procedure to a 1-s cue-target interval mostly reveals three lateralized components that are characterized by different topographies. The early directing attention negativity (EDAN) is a contralateral negativity with a maximum above occipito-parietal sites at approximately 200-400 ms after cue onset. This component is thought to reflect the first stage of attentional orienting by selectingthe relevant part of the attentional cue (see Van Velzen & Eimer, 2003). The second component, the anterior directing attention negativity(ADAN), is maximal above anterior sites around 400 ms after cue onset and is thought to reflect activity from premotor cortex and/or the frontal eye fields. Van der Lubbe et al. (2006) speculated that this component might actually reflect saccadic inhibition. It was reasoned that saccades commonly have to be suppressed during the cue-target interval because participants are instructed to do so. Moreover, they observed in their saccade-locked analysis a positive peak above the same anterior sites shortly before execution of the saccade, which might imply that the frontal negativity reflects inhibition of saccades. The third component is the late directing attention positivity (LDAP), being maximal above posterior sites around 500-700 ms after cue onset, which might reflect the final stage in which attention modulates activity along the ventral stream (Hopf & Mangun, 2000). According to Seiss, Gherri, Eardly, and Eimer (2007) both the ADAN and the LDAP are related to supramodal attentional control processes as these components are observed in auditory spatial attention tasks as well. In other studies, however, it was argued that the processes reflected in the ADAN operate within a somatotopic or anatomical reference frame, as polarity relative to cue direction reverses with crossed hands (Eardly & Van Velzen, 2011; Eimer, Forster, & Van Velzen, 2003), whereas the LDAP appears to be related to processes that operate within an external reference frame, independent from hand placement (but see Gherri & Forster, 2012).

Despite the high specificity of the ERL measure to changes in spatial attention this method is likely to have some shortcomings. As ERLs are derived from ERPs, they suffer from the same problem as ERPs (see Baar, Schürmann, Demiralp, Baar-Eroglu, & Ademoglu, 2001). ERLs like ERPs do not take dynamical changes in the brain’s intrinsic activity into account. The basic assumption of the averaging method is that effects are temporally related to external events such as a cue indicating the relevant side. However, although a part of the neuronal activity will be directly related to an external event, the brain is not a passive medium but a continuously active dynamic system containing numerous internal loops. Therefore, it seems likely that the majority of neuronal activity is internally generated, and not strictly bound to external events. Precisely, this activity is cancelled out by employing the normal averaging procedure (see Buzsáki, 2006). Herrmann, Grigutsch, and Busch (2005) also made a distinction between evoked oscillations on single EEG trials that are time-locked to specific events like stimulus onset, and induced oscillations that are produced by internal processes. Due to variations in the phase of the latter oscillations, averaging across a large number of trials will mainly reveal evoked oscillations, whereas activity from induced oscillations will get lost. This effect will be especially large in the case of higher spectra (like the γ-band) but it may already play a role for lower bands like β, α, θ and even (for a rough indication of the frequency ranges of these bands, see Table 1).

The new EEG measure for spatial attention that we want to propose here is the LPS. It builds further on the lateralization index on the basis of power as proposed by Thut et al. (2006) and can be applied after performing wavelet analyses (e.g., see Baar et al., 2001; Samar, Bopardikar, Rao, & Swartz, 1999), modified temporal spectral evolution (TSE), which is related to the event-related desynchronization/synchronization (ERD/ERS) method (see Thut et al., 2006), or after application of band pass filters.

(3) $$\mathrm{LPS}{\left(\omega_{p}\right)}_{t}=\left(\left(\text{left cues}\frac{\left(\omega_{\mathrm{pt}}\left(\mathrm{PO7}\right)–\omega_{\mathrm{pt}}\left(\mathrm{PO8}\right)\right)}{\left(\omega_{\mathrm{pt}}\left(\mathrm{PO7}\right)+\omega_{\mathrm{pt}}\left(\mathrm{PO8}\right)\right)}\right)+\left(\text{right cues}\frac{\left(\omega_{\mathrm{pt}}\left(\mathrm{PO8}\right)–\omega_{\mathrm{pt}}\left(\mathrm{PO7}\right)\right)}{\left(\omega_{\mathrm{pt}}\left(\mathrm{PO7}\right)+\omega_{\mathrm{pt}}\left(\mathrm{PO8}\right)\right)}\right)\right)/2$$

In this calculation, the power within a specific frequency band ω_p_ at time point *t* is determined for the hemispheres ipsilateral and contralateral to the direction of left and right cues. The ipsi-contralateral difference in power for each cue direction is scaled by the sum of activation of both hemispheres, similar to the procedure employed by Thut et al. (2006). The same calculation is performed for both cue directions after which the average of these estimates is determined. The latter procedure is in fact a double subtraction procedure, comparable to the method that is used to calculate the LRP or ERLs. Values of the LPS vary from -1 to +1. A positive sign indicates that the power within a specific frequency band ω was larger above the hemisphere ipsilateral to the cued side than contralateral, whereas a negative sign indicates the opposite pattern. A value of zero signifies the absence of hemispherical differences. The LPS index can be calculated for different spectra. As different frequency bands have been related to inhibition (α) and activation (γ), the meaning of a positive or negative value of this index will depend on the specific frequency band that was explored.^4^ The LPS procedure can also be applied on ERPs, which we denoted as the LPS-ERP. This computation seems relevant for the distinction of induced and evoked activity, as induced activity should not be present in the LPS-ERP (see below).

For several reasons, we think that the LPS may reveal important characteristics that will increase our understanding of the processes carried out when attention is being directed at a specific location. First, the calculation of power indices implies that variability in the onset of processes across trials has much less chance to result in a cancelation of activity as power is a measure of energy present in a signal (see Herrmann et al., 2005). Second, application of the double subtraction technique such as employed when computing ERLs and the LRP has the important advantage that general hemispherical differences unrelated to the direction of attention are cancelled out, making the index specifically sensitive to changes in spatial attention. Third, exploring various frequency bands (here limited from the lower θ band to the upper β band) may reveal new information that is likely to increase our understanding of spatial attention. Fourth, relating the LPS findings with ERLs, especially after additionally applying the LPS procedure on individual ERPs, the LPS-ERP, may additionally provide highly relevant information.

For example, Makeig et al. (2002) compared the power spectra of individual ERPs with raw EEG and observed similar topographies for both of them. Interestingly, they argued that their ERP features primarily arose from phase resetting of ongoing EEG processes, in line with the hypothesis by Klimesch (2011) and ideas of Baar (1980). In a recent paper by Grent-‘t-Jong, Boehler, Kenemans, and Woldorff (2011), a comparison was made between the biasing-related negati-vity (BRN; see ^Footnote 3^) and lateralized changes in power. They compared three task variants of the endogenous orienting paradigm, and argued on the basis of their results that the BRN reflects an influence on perceptual processes (i.e., attention affects perception), whereas the effects on power might point to changes in the task set, related to the presetting of stimulus-response (S-R) links (somewhat in line with the ideas of attention for action; e.g., Van der Heijden, 1992, 2004).

In the current paper, a comparison between the results of the LPS procedure on individual ERPs, the LPS-ERP, and the LPS on raw EEG may reveal whether specific processes are externally evoked by the cue or are more likely to be internally induced. For example, a clear LPS component within a specific time window in combination with comparable activity in the LPS-ERP analysis (regarding topography, timing, and spectral characteristics) points to externally evoked activity, whereas the absence of such activity suggests that this component is internally induced rather than externally generated.^5^ We expected to replicate previous ERL findings, and additionally, to observe comparable results of source localization analyses on the ERLs as in the study by Van der Lubbe et al. (2006) by employing the brain electric source algorithm (BESA) software. Regarding the LPS in the cue-target interval, for the α band we predicted to observe a positive value (i.e., increased ipsilateral vs. contralateral α power) just before target onset thereby replicating and extending the results mentioned in our introduction. Earlier effects are also likely to be present, for example, within the time windows in which ERL components have been observed. Apart from the α band, effects are likely to be present in other bands as well, therefore comparable effects were predicted for the β range. Finally, a comparison between ERLs and the LPS-ERP may reveal whether some evoked effects may not have shown up in ERLs due to individual differences. We restricted our statistical analyses to a subset of electrodes chosen on the basis of results of earlier studies (see the Method section).

## Method

### Participants

Twelve participants, all right-handed as assessed with Annett’s Handedness Inventory (Annett, 1970), took part in the experiment. They were recruited from the local student population at the University of Twente, and consisted of four women and eight males (*M*_age_ = 20 years, ranging from 18 to 24 years). All participants had normal or corrected-to-normal vision, were not color blind, and had no history of neurological diseases. They signed informed consent at the start of the experiment. The participants served as a control group for a study on dyslexia, therefore several tests were carried out (a dyslexia screening test [DST^NL^], the Trail Making Test, the Bourdon-Wiersma Test, and the Balloons Test). The latter acquired data are beyond our current interest, and therefore will not be reported here. The employed procedures were approved of by a local ethics committee at the Faculty of Behavioral Sciences.

### Task and stimuli

**Figure 1.**
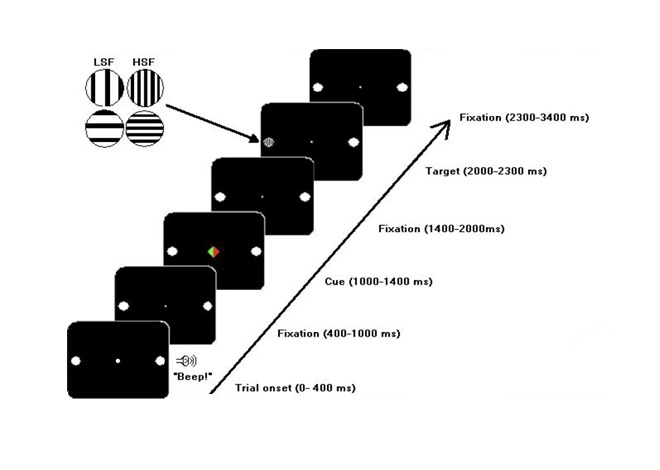
The sequence of events in a trial. Four types of targets were used; they had either low or high spatial frequencies (LSF or HSF) and had a vertical or horizontal orientation. Left or right button presses depended on target orientation.

A variant of the Posner (1980) endogenous spatial cuing paradigm was used comparable to the discrimination task used by Van der Lubbe et al. (2006). A default display consisted of a centrally presented white fixation point (0.164° × 0.164°) on a black background, accompanied by two open light-grey circles at the left and right side of the screen (at 12.06° with *r* = 0.614°; see Figure 1). Onset of a trial was marked by a short auditory warning stimulus and a slight enlargement of the fixation dot for 400 ms. At trial onset, participants were instructed to direct their eyes towards the fixation point. After presenting the default display for another 600 ms, a diamond-shaped cue, consisting of two colored triangles (red and green, with one color defined as relevant) pointing to the left and right circles, was displayed for 400 ms. This cue was replaced by the default display for another 600 ms. At 1,000 ms after cue onset, a target was presented for 300 ms in either the left or the right circle. This was either a vertically or a horizontally striped target with either two thick lines (0.25°; low spatial frequency) or six thin lines (0.082°; high spatial frequency). Horizontally striped targets required a left button response, and vertically striped targets required a right response. Spatial frequency itself was irrelevant to the required response, but was thought to result in relatively easy (with low spatial frequency) and hard to be discriminated stimulus orientations. Responses were to be made as fast and accurately as possible. After target-offset, the default display was presented for another 1,100 ms until a new trial began.

The relevant color cue (the red or the green triangular part of the diamond) differed between two sessions that each contained two blocks, and the order of these sessions was counterbalanced between participants. The direction of the relevant color cue accurately predicted the target location on 80% of the trials (validly cued trials), but on 15% of the trials, the target was displayed at the opposite location (invalidly cued trials). On the remaining 5% of the trials, no target was presented (catch trials). The task consisted in total of 672 experimental trials, which were divided between four blocks of 168 trials each. Each block started with 20 practice trials. Execution of the whole task took approximately 70 min.

### Apparatus and EEG recordings

The participants sat on a comfortable chair in a darkened room at approximately 70 cm in front of a 17”-CRT-screen. Stimuli were presented by using Presentation software (Neurobehavioral Systems, Inc., 2012) installed on a separate experimental computer. Required left and right button presses with the left and right index fingers were to be made by pressing the left or right “Ctrl” key on a standard QWERTY keyboard.

Passive Ag/AgCl ring-electrodes were placed according to the extended 10-20 system at 61 locations. The electrodes were mounted in an elastic cap (Braincap, Brainproducts GmbH). The horizontal and vertical electro-oculogram (hEOG and vEOG) were recorded together with the EEG by applying electrodes above and below the left eye and by applying electrodes at the outer canthi of both eyes. After applying electrode gel and using standard procedures to improve conductivity, electrode resistance could be kept below 5 k. A 72-channels QuickAmp (Brain Products GmbH) amplifier was used to amplify the EEG and EOG. This amplifier has a built-in average reference. Together, EEG, EOG, and task-related events such as stimulus onset and responses were registered with BrainVision Recorder (BrainProducts GmbH) installed on a separate acquisition computer. Signals were sampled at a rate of 500 Hz with the following online filters: TC = 5 s, low pass filter 100 Hz, notch-filter 50 Hz.

### Data processing

Processing of the data was carried out with Brain Vision Analyzer 2.0 (Brain Products GmbH, 2012). The data were first partitioned in segments from -500 to 2,500 ms relative to cue onset, with a baseline set from -100 to 0 ms. Horizontal and vertical movements of the eyes were marked when amplitudes on the hEOG and vEOG channels exceeded the values of +/-40 μV and +/-120 μV, respectively. Earlier studies showed that a value of +/-40 μV corresponds with a horizontal eye movement of approximately 2° (e.g., see Van der Lubbe & Woestenburg, 1997). Subsequently, those trials in which eye movements were detected from 0 to 1,000 ms relative to cue onset were removed. This procedure left on average 75% of the trials. This rigorous procedure was carried out to exclude the possibility that the effects of cue validity on our behavioral measures may be due to overt rather than covert orienting. Furthermore, this procedure controlled for the possibility that observed effects in the cue-target interval were unrelated to saccade execution.

Only trials without detectable eye movements and button presses in the cue-target interval were used for the reaction time (RT) and proportion of correct (PC) analyses. Individual averages on RT (> 100 and < 2,000 ms) and PC were determined as a function of Cue Validity (validly or invalidly cued targets), Spatial Frequency of the target (low or high), Target Side (LVF or RVF), and Target Orientation (horizontal or vertical). These factors were analyzed by employing a repeated measures analyses of variance (ANOVA).

All remaining segments were used for an Independent Component Analysis (ICA, Infomax) to remove residual activity due to horizontal or vertical eye movements. This method seems superior to a range of other methods (e.g., see Jung et al., 2000).

#### EEG analyses of the cue-target interval

Segments were selected that contained no responses (i.e., no saccades and/or no button presses) in the cue-target interval. EEG channels containing artifacts were removed (gradient criterion: 100 μV per 1 ms, min-max criterion: -/+ 150 μV, low activity criterion: 0.1 μV for 50 ms; individual channel mode). The number of removed channels was in general very low (< 2%). The only exception was one participant who had one EEG channel for which 15.3% of the trials had to be removed. In a first analysis, ERPs were computed for left and right cues. Next, the double subtraction technique (see Formula 2) was applied to compute ERLs for 26 symmetrical electrode pairs. To evaluate the whole time range from 200 till 1,000 ms after cue onset with 20-ms time windows on a series of preselected electrodes (see below), we implemented the Benjamini and Hochberg (1995) procedure. This procedure limits the false discover rate (FDR) in the case of multiple comparisons, which was advocated by Lage-Castellanos, Martínez-Montes, Hernández-Cabrera, and Galán (2010) and by Groppe, Urbach, and Kutas (2011). Given the previous findings by Lasaponara, Chica, Lecce, Lupianez, and Doricchi (2011); Van Velzen and Eimer (2003); Praamstra and Kourtis (2010), and by Van der Lubbe, Jakowski, and Verleger (2005), we limit-ed the statistical analyses to the following electrode pairs: F5/F6, FC5/FC6, P3/P4, PO3/PO4, PO7/PO8, and O1/O2. As there are 40 *t*-tests per electrode pair, this amounts to a total of 240 tests. The obtained *p* values (one-tailed, as a deviation from zero indicates relevant activity) based on absolute *t*-values were subsequently ranked from smallest till largest (1, 2, …, *k*, …, 240). Next, those values were considered as significant when the observed *p* value was smaller or equal to *t*_crit_, which was defined as (*k*/240) × 0.05. This implied that the significance criterion for the smallest *p* value was .00021, next .00042, .00063, and so on, until finally reaching the criterion of .05. We additionally checked with the hEOG whether small saccades below the critical threshold related to the cued side (polarity was inverted in the case of left cues) remained that might account for observed ERL effects.

In an earlier paper, the likely sources of observed ERLs were estimated by employing BESA (Van der Lubbe et al., 2006). We again applied BESA (now version 5.1.6) to determine the likely sources of the observed lateralized activities, to estimate how the contribution of these sources changed over time, and to compare these results with the previous study.

In the second analysis, a wavelet analysis was carried out on single trials on all EEG channels. A Complex Morlet wavelet (*c* = 5) was chosen (e.g., see Freunberger et al., 2008; Gladwin, Lindsen, & De Jong, 2006). Furthermore, we chose segments from -500 till 2,500 ms relative to cue onset, which allowed the reliable assessment of power of frequencies starting from the lower θ band (> 4 Hz). The minimum and maximum frequencies were set at 4 and 20 Hz. This frequency range was separated in seven logarithmic steps, resulting in bands covering the lower θ to upper β range (see Table 1). Gabor normalization was employed. After determining the absolute power of specific frequencies at different points in time per trial, averages for left and right cues were computed per individual. Next, lateralization indices were calculated for the different frequency bands for left and right cues.

**Table 1.** The Different Frequency Bands With Their Central Frequencies and Borders (1 *SD*) in the Current Paper Extracted by Employing Wavelet Analyses

Band	Central frequency (Hz)	Lower band (Hz)	Upper band (Hz)	Associatedband
1	4	3.2	4.8	θ_1_
2	5.2	4.2	6.3	θ_2_
3	6.8	5.5	8.2	θ_3_
4	8.9	7.2	10.7	α_1_
5	11.7	9.4	14.0	α_2_
6	15.3	12.2	18.4	β_1_
7	20	16	24	β_2_

(4) $$\text{Index left cues}=\frac{\left(\omega_{\mathrm{pt}}\left(\mathrm{PO7}\right)–\omega_{\mathrm{pt}}\left(\mathrm{PO8}\right)\right)}{\left(\omega_{\mathrm{pt}}\left(\mathrm{PO7}\right)+\omega_{\mathrm{pt}}\left(\mathrm{PO8}\right)\right)}\;\text{Index right cues}=\frac{\left(\omega_{\mathrm{pt}}\left(\mathrm{PO8}\right)–\omega_{\mathrm{pt}}\left(\mathrm{PO7}\right)\right)}{\left(\omega_{\mathrm{pt}}\left(\mathrm{PO7}\right)+\omega_{\mathrm{pt}}\left(\mathrm{PO8}\right)\right)}$$

These power indices were computed for all symmetrical electrode pairs. Furthermore, an average was computed across both indices (see Formula 3), thereby constructing the LPS for 26 symmetrical electrode pairs. These results were again evaluated per band by using the above-mentioned procedure for the same preselected set of electrodes as in the case of the ERLs from 200 to 1,000 ms after cue onset.

Finally, the LPS procedure as described above was applied on the ERPs for left and right cues thereby providing us with the LPS-ERP. This analysis allows us to establish whether observed LPS components in the second analysis are more likely to have an induced rather than an evoked nature, that is, when they are present in the LPS results and not in the LPS-ERP results. Furthermore, the LPS-ERP results might reveal effects within specific time windows with certain topographies that do not show up in ERLs due to individual differences.

## Results

### Behavioral measures

Responses were faster on validly cued than on invalidly cued trials (742 vs. 799 ms), *F*(1, 11) = 24.2, *p* < .001. Responses were slower for targets with a high spatial frequency than for targets with a low spatial frequency (820 vs. 720 ms), *F*(1, 11) = 19.2, *p* < .001. In addition, targets with a horizontal orientation were discriminated faster (745 ms) than targets with a vertical orientation (795 ms), *F*(1, 11) = 21.9, *p* < .001. A just significant interaction was observed between Spatial Frequency, Cue Validity, Target Orientation, and Target Side, *F*(1, 11) = 5.3, *p* < .05.

Separate analyses for LVF targets showed main effects of spatial frequency (low: 716 ms, high: 813 ms), target orientation (horizontal: 738 ms, vertical: 791 ms), and cue validity (valid: 739 ms, invalid: 790 ms), *F*(1, 11) > 7.0, p < .03. Separate analyses for RVF targets revealed main effects of spatial frequency (725 vs. 827 ms) and cue validity (745 vs. 807 ms), *F*(1, 11) > 14.2, *p* < .005, a just non-significant effect of target orientation, *F*(1, 11) = 4.8, *p* = .052, and a significant interaction between Spatial Frequency, Cue Validity, and Target Orientation, *F*(1, 11) = 5.7, *p* < .04. Separate analyses for low spatial frequency targets only revealed an effect of cue validity (696 vs. 753 ms), *F*(1, 11) = 15.4, *p* < .005, whereas separate analyses for high frequency targets revealed both effects of cue validity (793 vs. 861 ms), *F*(1, 11) = 6.5, *p* < .03, and target orientation (horizontal: 785, vertical: 870 ms), *F*(1, 11) = 10.0, *p* < .01.

In line with the previous findings, analyses on the accuracy of responses revealed that PCs were smaller for high spatial frequency than for low spatial frequency targets (70.3 vs. 90.8%), *F*(1, 11) = 28.4, *p* < .001. An interaction was observed between Target Orientation, Cue Validity, and Target Side, *F*(1, 11) = 11.1, *p* < .01.

Separate analyses for LVF targets only revealed an effect of spatial frequency, *F*(1, 11) = 19.1, *p* < .002, with better performance for low than for high frequency targets (90.9 vs. 71.3%). Separate analyses for RVF targets revealed an effect of spatial frequency, *F*(1, 11) = 19.1, *p* < .002, a main effect of cue validity, *F*(1, 11) = 5.2, *p* < .05, and an interaction between Target Orientation and Cue Validity, *F*(1, 11) = 6.3,p < .03, which reflected an effect of cue validity for horizontally oriented targets (valid: 86.4%, invalid: 78.7%), but no such effect for vertically oriented targets (77.7 vs. 77.8%).

### EEG analyses of the cue-target interval

**Figure 2.**
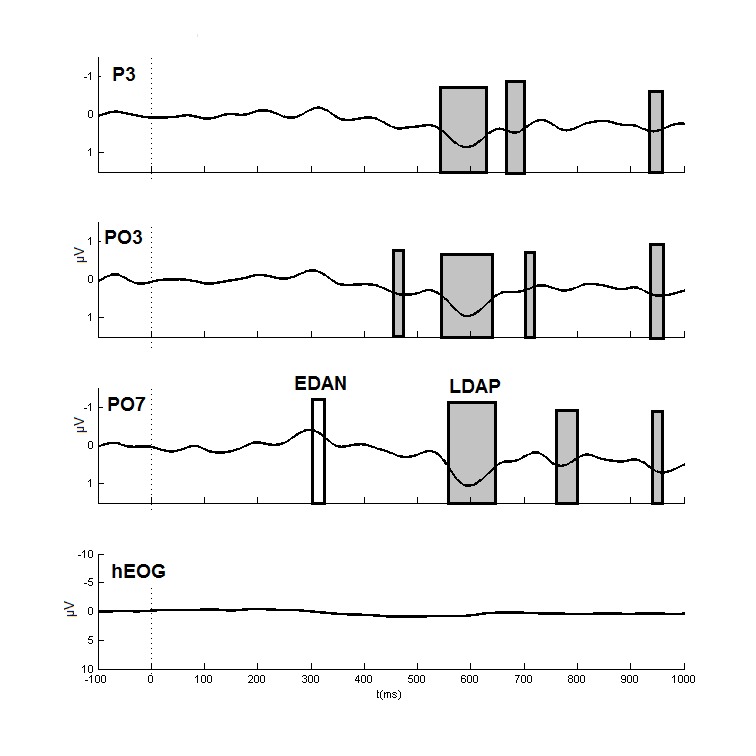
Grand average event-related lateralizations (filtered for display purposes with a low pass filter of 12 Hz, 24 dB/oct) and cue-direction specific hEOG as observed during the cue-target interval at parietal, occipito-parietal, and occipito-temporal sites. In our labeling of the channels, we indicated the locations on the left hemisphere but observed potentials are the result of double subtractions (see the Method section). The time windows in which significant effects were observed after application of the Benjamini and Hochberg (1995) procedure are indicated in gray boxes. The early directing attention negativity (EDAN) was significant at O1 (see Table 2), which is not displayed here. The time interval in which this occurred is indicated for PO7 with the light gray box. LDLDAP = late directing attention positivity.

**Figure 3.**
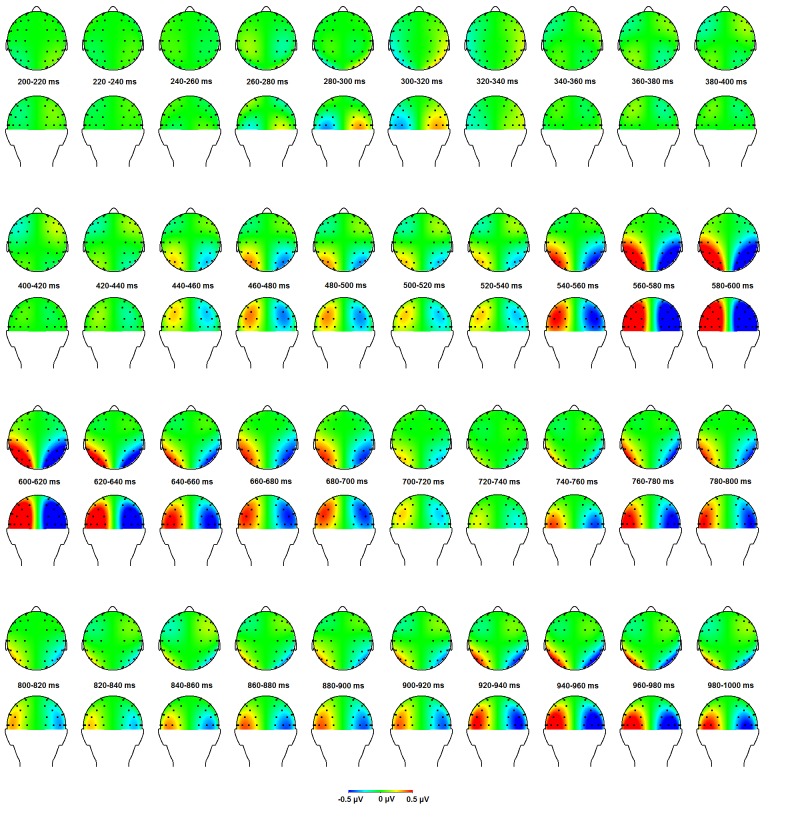
Topographical maps of the event-related lateralizations in 20-ms windows from 200 to 1,000 ms after cue onset based on interpolation of spherical splines (fourth order). In the left hemisphere, the contra-ipsilateral difference map is displayed, whereas an inverted ipsi-contralateral difference map is presented for the right hemisphere.

ERLs for a selection of relevant electrodes accompanied with topographical maps at relevant time intervals are displayed in Figures 2 and 3. Analyses were performed on 20-ms intervals from 200 to 1,000 ms after cue onset for the selected electrode pairs (see above). A summary of the most relevant findings on the ERLs is presented in Table 2. Application of the Benjamini and Hochberg (1995) procedure revealed that the critical t-value for the ERLs amounted to 3.1 (*p* = .0049). The presence of the EDAN was confirmed from 300 to 320 ms, no ADAN seemed present, but a highly pronounced LDAP was visible from 540 until at least 640 ms, and this effect returned shortly before target onset, from 940 to 960 ms after cue onset. We decided to denote the latter positivity, the biasing related positivity (BRP), comparable to the term used by Grent-‘t-Jong et al. (2011). Interesting is that effects seems slightly more pronounced above occipito-parietal than occipito-temporal sites. For all observed effects we examined whether there was possibly a relation with small eye movements related to the cued side. First, inspection of each individual participant showed that amplitudes in the whole cue-target never exceeded a value of 1.7 *V* (*SD* = 0.8), suggesting that the exclusion criteria were effective. Nearly all correlations between hEOG and the relevant EEG channels were non-significant (*p* > .20), however, a significant correlation between P3 and hEOG was observed from 560-580 ms (*p* = .028, *r*_2_ = .63). This observation suggests that the parietal focus may partially reflect the execution of very small below threshold saccades. Importantly, the great majority of observed effects appears to have an attentional nature.

**Table 2.** A Summary of Effects Observed on the Event-Related Lateralizations

ERL			
Component	Window	Maxima	*t*(11)
EDAN	300-320	O1	-3.8*
	460-480	PO3	3.6*
LDAP	540-620	P3	3.4*- 5.4***
	540-640	PO3	3.1*- 6.0***
	560-640	PO7	3.2*- 4.4***
	580-620	O1	3.2*- 3.3*
	660-700	P3	4.0**- 4.1**
	700-720	PO3	5.2***
	760-800	PO7	3.1*- 3.7*
BRP	940-960	P3	4.5***
	940-960	PO3	3.7*
	940-960	PO7	3.4*

*Note*. Effects are described in terms of contra-ipsilateral differences, which in the displayed topographies are projected on the left hemisphere (therefore O1, PO3, PO7, etc.). BRP = biasing related positivity. EDAN = early directing attention negativity. ERL = event related lateralization. LDAP = late directing attention positivity. *p < .005. **p < .001. ***p < .0005 (one-tailed).

**Figure 4.**
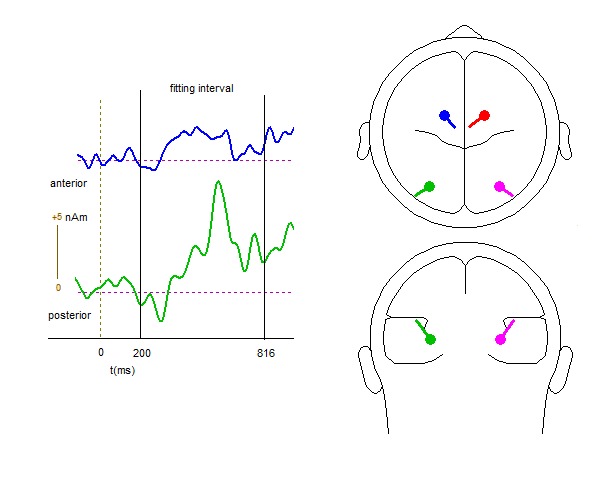
Results of source analysis with BESA on the event-related lateralizations observed in the cue-target interval. Estimated activity of the posterior (green) and the anterior source (blue) is indicated in the left panel.

A principal component analyses (PCA, implemented in BESA) on the ERLs (filtered with a lowpass filter of 12 Hz, 24 dB/oct) showed that two principal components could account for nearly all the variance for the interval from 200 to 816 ms. The first component accounted for 89.7% and the second component accounted for 5.7% of the variance. On the basis of this result a model was chosen with two symmetrical dipole pairs (location symmetric and orientation fixed) as activity in one hemisphere is inverted in the other hemisphere (see also Van der Lubbe et al., 2006). After the fitting procedure, residual variance of the source model amounted to 7.7%. A posterior, possibly occipital source (*x* = 32, *y* = -49, *z* = 34) and an anterior, fronto-central source (*x* = 18, *y* = 15, *z* = 93.6) were found (see Figure 4). Estimated activity of these sources over time suggests that initially at around 300 ms, the posterior source is shortly active, which may reflect the EDAN. At around 480 ms after cue onset, the anterior source became active, which might reflect activity from the frontal eye field (FEF), although no support for this was observed in our ERL analyses. Next, a strong burst of activity was present for the posterior source at around 500 ms after cue onset, which remains active until the end of the fitting interval, which may reflect the LDAP and the BRP. This pattern of observed activities is quite comparable to those estimated in the experiments reported by Van der Lubbe et al. (2006).

Analyses on the LPS in the aforementioned frequency bands revealed that none of the performed *t*-tests crossed the criterion value of 4.98 (*p* < .00021). We decided to report results when the criterion of *t* = 3.1 (the same criterion as reached for the ERLs) was crossed for at least two successive time windows (for a comparable procedure, see Talsma, Wijers, Klaver, & Mulder, 2001) as we have the impression that the employed procedure to minimize the FDR was too conservative (see Table 3).

**Table 3.** A Summary of the Results for the LPS on the raw EEG When the Significance Criterion Was Crossed for at Least Two Successive Time Windows

Wavelets		LPS		Related ERL component
Band	Window (ms)	Maxima	*t*(11)	
θ_2_	260-460	F6	3.1*- 3.8*	-
	580-720	PO8	3.3*- 3.7*	LDAP
	660-740	PO4	3.1*- 3.3*	LDAP
	680-780	P4	3.1*- 3.5*	LDAP/BRP
θ_3_	420-620	PO8	3.2*- 4.3**	LDAP
	440-520	O2	3.2*- 3.4*	LDAP
	600-700	P4	3.2*- 3.7*	LDAP
α_1_	540-580	PO8	3.1*	LDAP
	920-1.000	PO8	3.2*- 3.9*	BRP
α_2_	420-580	PO8	3.2*- 3.4*	LDAP
β_1_	420-460	PO8	3.5*- 3.7	LDAP
	620-660	F6	3.4*- 3.5*	-
	680-720	FC6	3.3*- 3.4*	-
β_2_	620-660	F6	3.3*- 3.6*	-

*Note*. Effects are described in terms of ipsi-contralateral differences (there- fore O2, PO4, PO8, etc.). We also indicated to what ERL component the specific activity seems to be related. BRP = biasing related positivity. EDAN = early directing attention negativity. ERL = event related lateralization. LDAP = late directing attention positivity. LPS = the lateralized power spectra. *p < .005. **p < .001. ***p < .0005 (one-tailed).

**Figure 5.**
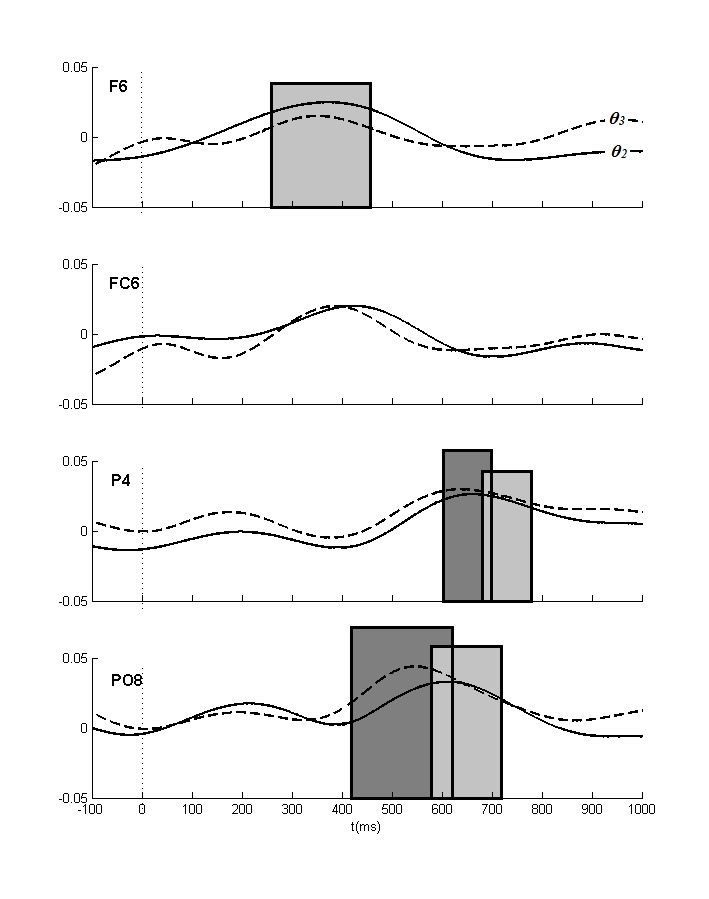
The lateralized power spectra for the θ_2_ and the θ_3_ bands. Positive values mean increased ipsilateral relative to contralateral power. The initial frontal effect (see Table 3) and the later posterior effects thus both reflect increased ipsilateral as compared to contralateral power. Significant effects (at least two successive windows *p* < .01) for the θ_2_ and the θ_3_ bands are indicated in light and dark gray boxes, respectively.

**Figure 6.**
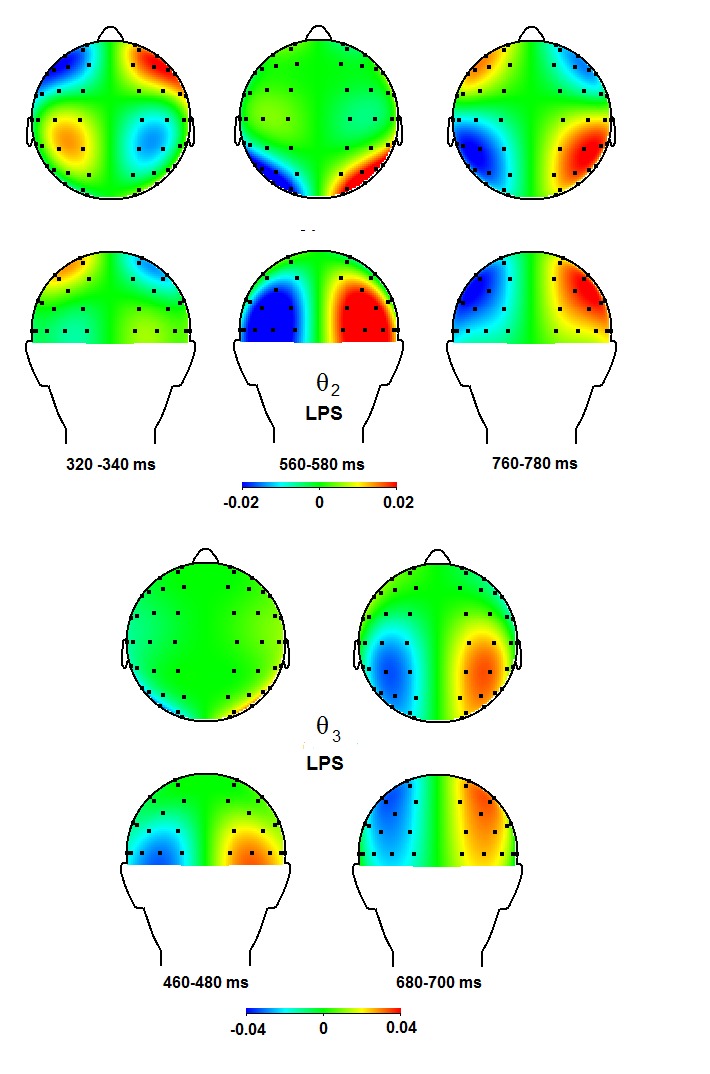
Topographical maps for the θ_2_ and the θ_3_ bands for time windows in which significant effects were observed, in the left and right panel, respectively. The left hemisphere reflects the contra-ipsilateral power difference, whereas the right hemisphere displays the ipsi-contralateral power difference. Positive values in the right hemisphere thus mean increased ipsilateral as compared to contra- lateral power. LPS = lateralized power spectra.

**Figure 7.**
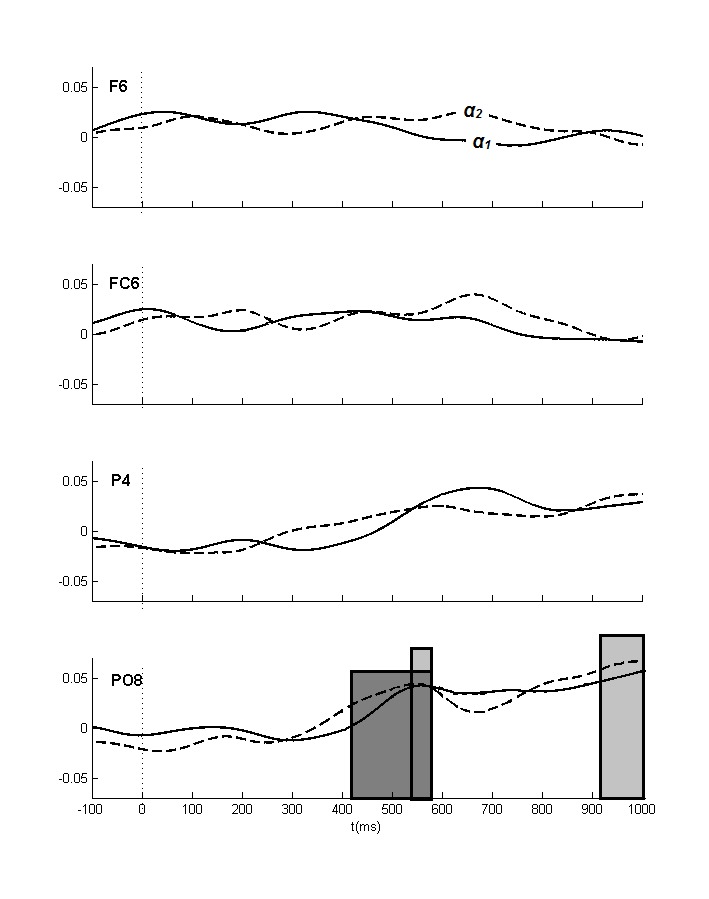
The lateralized power spectra for the α_1_ and the α_2_ bands. Positive values mean increased ipsilateral relative to contralateral power. The occipital effect (see Table 3) for the α_1_ and the α_2_ band thus both reflect increased ipsilateral as compared to contralateral power. Significant effects (at least two successive windows *p* < .01) for the α_1_ and the α_2_ bands are indicated in light and dark gray boxes, respectively.

**Figure 8.**
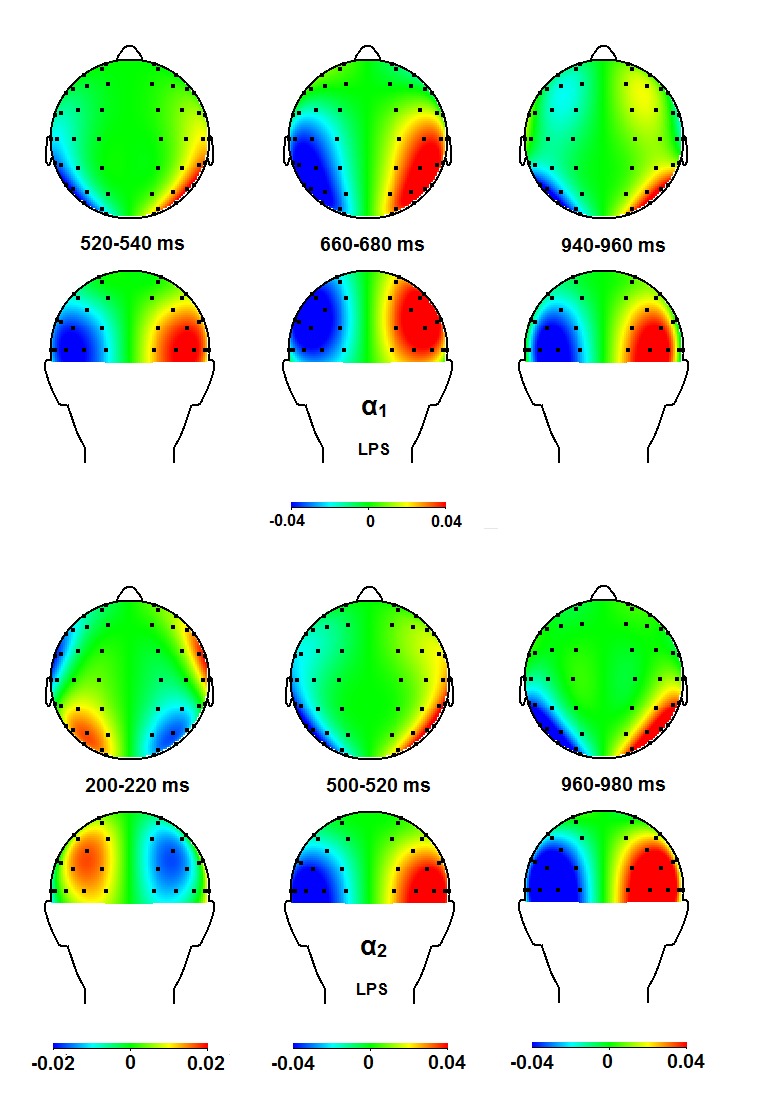
Topographical maps for the α_1_ and the α_2_ bands for time windows in which significant effects were observed, in the upper and lower panel, respectively. For further descriptions, see Figure 6. LPS = lateralized power spectra.

**Figure 9.**
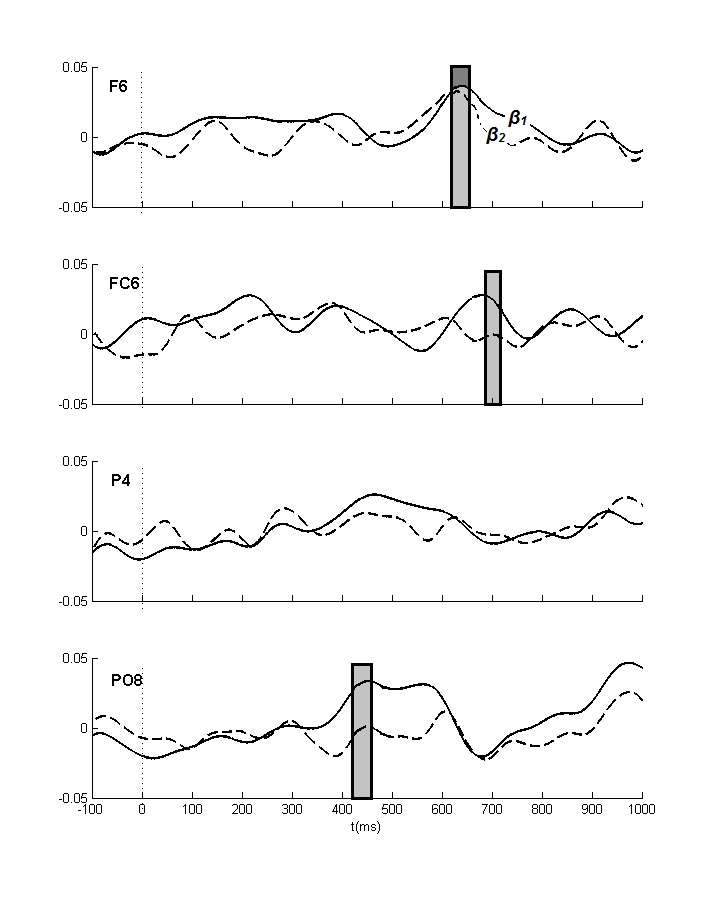
The lateralized power spectra for the β_1_ and the β_2_ bands. Positive values mean increased ipsilateral relative to contralateral power. Significant effects (at least two successive windows *p* < .01) for the β_1_ and the β_2_ bands are indicated in light and dark gray boxes, respectively.

**Figure 10.**
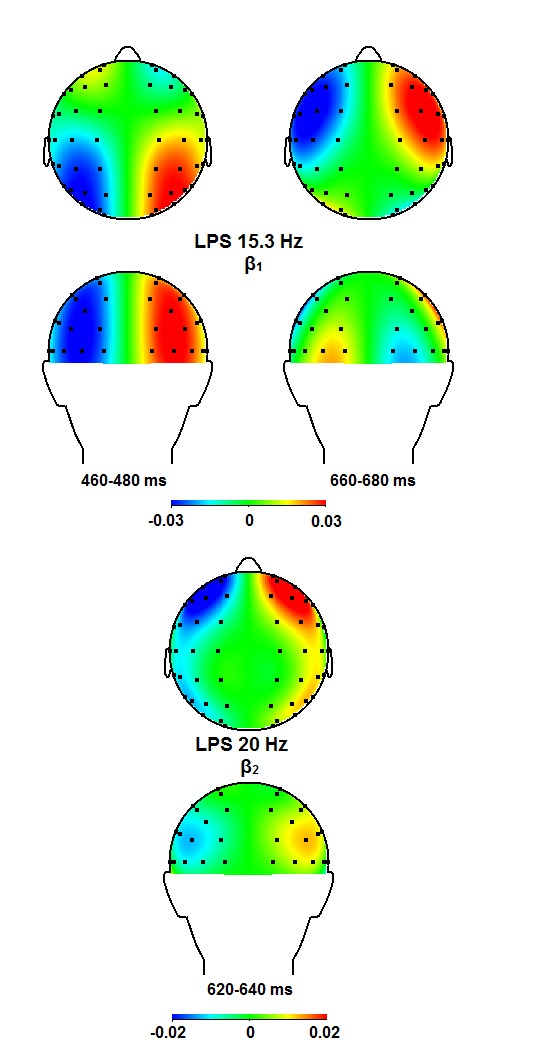
Topographical maps for the β_1_ and the β_2_ bands for time windows in which significant effects were observed. For further descriptions, see Figure 6. LPS = lateralized power spectra.

No effects were obtained for the lowest θ_1_ band. For the θ_2_ band (see Figures 5 and 6), increased ipsilateral anterior power (260-460 ms)is followed by an occipital focus (580-720 ms), which thereafter shifts to parietal sites (680-780 ms). The latter shift is also visible in the higher θ_3_ band, where an initial posterior focus (420-620 ms) is followed by a more parietal focus (600-700 ms). In the lower α_1_ band, there is support for an initial occipital focus (540-580 ms) that disappears and returns just before target onset (920-1,000 ms). In the upper α_2_ band, an effect is present above occipital sites from 420 to 580 ms (see Figures 7 and 8).Finally, in the lower β_1_ band (see Figures 9 and 10), an initial occipital focus (420-460 ms) was followed by an anterior focus (620-720 ms), whereas in the upper β_2_ band, an anterior focus is present from 620 to 660 ms.

**Figure 11.**
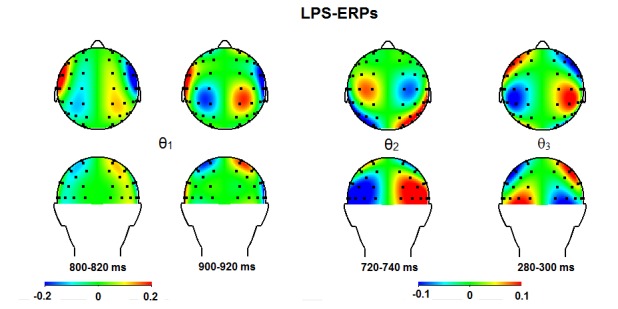
Topographical maps for the θ_1_, the θ_2_, and the θ_3_ bands for time windows in which significant effects were observed (see Table 4) after performing the lateralized power spectra analysis on the event related potentials (LPS-ERP). Ipsi-contralateral estimates are projected on the right hemisphere.

**Figure 12.**
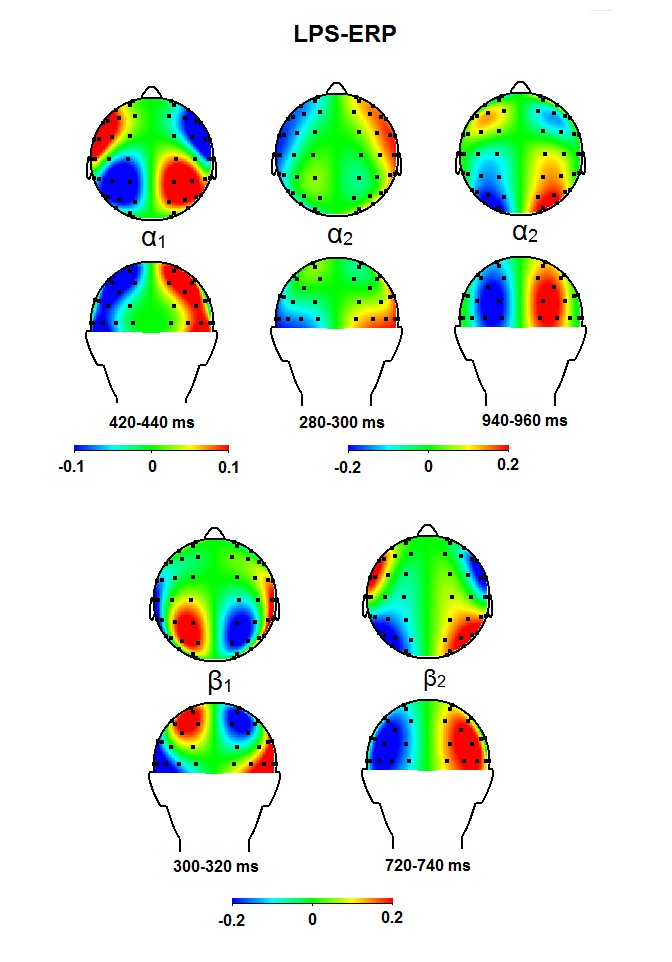
Topographical maps for the α_1_, the α_2_, the β_1_, and the the β_2_ band for time windows in which significant effects were observed (see Table 4) after performing the lateralized power spectra analysis on the event related potentials (LPS-ERP). Ipsi-contralateral estimates are projected on the right hemisphere.

The statistical results of the LPS on the individual ERPs also did not cross the criterion *t*-value of 4.98. As these analyses were performed to determine whether effects present in the LPS on the raw EEG can be considered as stimulus-evoked (then effects should also be present on the LPS-ERP) or induced (then no effects should be visible in the LPS-ERP) we decided to apply a more liberal criterion. We reported results when activity in a single time window crossed the criterion *t*-value of 3.1. Results of these analyses are displayed in Table 4. Relevant topographies are displayed in Figures 11-12. In the θ_1_ band, decreased ipsilateral power on anterior sites was observed from 740 to 840, which returned from 860 to 940 ms (see Figure 11). In the θ_2_ band, posterior effects were found from 660 to 760 ms, reflecting increased ipsilateral vs. contralateral power. In the θ_3_ band, we observed a short early decrease of ipsilateral power at anterior sites. No other effects were observed in the θ_3_ band. In the α_1_ band, an anterior effect was observed from 400 to 440 ms, showing decreased ipsilateral versus contralateral power (see Figure 12). In the α_2_ band, we noticed an early posterior effect from 280 to 300 ms, and a later posterior effect from 940 to 960 ms, both signaling increased ipsilateral vs. contralateral power. In the β_1_ band, we noticed an early parietal effect from 300 to 320 ms, reflecting decreased ipsilateral vs. contralateral power. Finally, in the β_2_ band, we noticed a late anterior effect, again decreased ipsi-lateral power, from 720 to 740 ms.

**Table 4.** A Summary of the Results of the LPS Analyses Performed on the Individual ERPs, the LPS-ERP, When the Significance Criterion Was Crossed

	LPS-ERP			Related ERL component
Band	Window (ms)	Maxima	*t*(11)	
θ_1_	740-840	FC6	3.1*- 3.2*	-
	860-940	FC6	3.1*- 3.7*	-
θ_2_	660-760	PO4	3.2*- 4.1*	LDAP
θ_3_	280-300	F6	3.2*	-
α_1_	400-440	FC6	3.3*	ADAN
α_2_	280-300	O2	3.5*	EDAN
	940-960	PO8	3.4*	BRP
	940-960	O2	3.3*	BRP
β_1_	300-320	P4	3.3*	EDAN
β_2_	720-740	FC6	3.2*	-

*Note*. Results of these analyses concern ipsi-contralateral differences (there- fore O2, PO8, P4, etc.). ADAN = anterior directing attention negativity. BRP = biasing related positivity. EDAN = early directing attention negativity. LPS-ERP = lateralized power spectra on ERPs. LDAP = late directing attention positivity. *p < .005. **p < .001. ***p < .0005 (one-tailed).

## Discussion

In our introduction, we argued that for the examination of attentional orienting in the Posner (1980) endogenous cuing paradigm, the focus on measures derived from ERPs that extract direction-related activity, such as the ERL, may possibly be less fortunate. Namely, the onset of a process like attentional orienting probably varies over trials, and in the case of higher spectra this varying activity will be subtracted out by the standard averaging technique. Therefore, an alternative measure was developed along the lines of the ERLs, again being specific to the locus of interest, but now based on the results of wavelet analyses, which incorporates the trial-to-trial variation: the LPS. This measure was determined for various frequency bands and may provide highly specific and new information about attentional orienting. Furthermore, performing the same analysis on ERPs, the LPS-ERP, may reveal whether observed effects can be considered as evoked (then effects should be visible in the LPS-ERP results) or rather as induced (i.e., when effects are only visible in the LPS; see Herrmann et al., 2005). Finally, comparing LPS-ERP results with ERLs may reveal whether some evoked effects may have been cancelled out for the reported ERL results due to individual differences.

Behavioral data confirmed major effects of attentional orienting by reducing response speed for attended as compared to initially unattended targets presented in the lateral LVF and RVF. Two different target types were employed (low/high spatial frequency), intermixed across trials, which had a large impact on performance; responses were slower and less accurate for high as compared to low spatial frequency targets. These data indicate that our paradigm was effective in manipulating visuospatial attention and also that target discrimination was rather difficult, thereby motivating participants to use the cues.

A double subtraction technique was applied on ERPs for the cue-target interval to construct the ERLs (see Figures 2 and 3). An EDAN was observed from 300 till 320 ms, being maximal at occipital sites (see Figure 3). This observed lateralization replicates previous findings (Van der Lubbe et al., 2006). The observed topography of the EDAN may accord with a modulation along the early part of the ventral visual pathway (see Van der Lubbe, Abrahamse, & De Kleine, 2012; see also Talsma, Mulckhuyse, Slagter, & Theeuwes, 2007). In a recent paper, Praamstra and Kourtis (2010) noticed that their EDAN had a more occipito-parietal distribution, which led them to conclude that the EDAN cannot be equated with the N2pc (or posterior contralateral negativity [PCN]). This observation contrasts with the interpretation of Van Velzen and Eimer (2003), who argued that the EDAN reflects directing of attention towards the relevant side of the cue (an N2pc in disguise). However, our source analyses (see Figure 4) additionally support a source in occipital rather than parietal cortex, in line with the view of Van Velzen and Eimer, and may indeed reflect attentional selection of the relevant part of the cue.

Examination of the LPS around the time range of the EDAN with a comparable topography and timing points to the θ_3_ (420-620 ms; Figures 5 and 6), the θ_2_ (420-580 ms; Figures 7 and 8), and the β_1_ (420-460 ms; Figures 9 and 10) bands, but these activities seem far too late to account for the EDAN. Examination of the LPS-ERP displays posterior effects in the α_2_ (280-300 ms) and the β_1_ bands (300-320 ms), which can very well account for the observed EDAN, although the parietal locus of the β_1_ band seems less appropriate. Thus, the EDAN, which can be considered as evoked activity as it shows up in our ERLs, was not visible in the LPS. A likely reason why it may be more difficult to observe effects on the LPS than on ERLs is that the LPS is more sensitive to noise as it cannot distinguish between signal and noise that have comparable spectral characteristics. As the EDAN may be characterized as posterior increased ipsilateral vs. contralateral power in the α_2_ band (see Figure 12), it may be argued that the EDAN reflects ipsilateral inhibition and/or contralateral disinhibition.

Unlike previous studies (e.g., Van der Lubbe et al., 2006) no ADAN was visible in our ERL data. It might be that the process reflected by the ADAN has a more induced nature than the EDAN and therefore does not necessarily show up in ERLs. If we focus on the results of our source analysis (Figure 4), then some frontal activity seems present, but no sign of this is visible in our topographic maps (Figure 3). If we look at our LPS results (Table 3) within the same time range as the activity of our frontal source (400-600 ms) with an anterior topography, then θ_2_ (240-460 ms), β_1_ (620-660 ms), and β_2_ (620-660 ms) might be candidates for the involved frequency bands, and all of them suggest increased ipsilateral as compared to contralateral power. Importantly, however, the LPS-ERP results (Table 4) show frontal activity in the α_1_ band from 400 to 440 ms, and later on there is as well activity in the θ_1_ band from 740 to 780 and from 800 to 940 ms (see Figure 11), and also in the β_2_ band from 720-740 ms. The latter activities seem a bit too late to account for the ADAN. Based on these findings, it may be argued that the ADAN has an evoked rather than an induced nature, but does not show up in the current ERLs due to individual differences. Regarding the anterior effects on the LPS in the higher β bands from 620 to 720 ms, some comparable effects seem present in the LPS-ERP results. Therefore, it may be argued that these activities also have an evoked nature, although it seems that they do not contribute to the ADAN as the involved frequency seems to high. On the basis of the review of Corbetta and Shulman (2002), it may be proposed that these different frontal activities originate from the FEF (see also Grent-‘t-Jong & Woldorff, 2007; Noudoost, Chang, Steinmetz, & Moore, 2010). In terms of the view of Grent-‘t-Jong and Woldorff this activity in the FEF might be the start of the cascade of attentional processes that subsequently affect parietal and occipital areas. Based on the results of Capotosto, Babiloni, Romani, and Corbetta (2009) it may even be argued that this concerns a causal influence, as they observed a disruption of posterior activity together with deficits in visual discrimination after the application of repetitive transcranial magnetic stimulation (TMS) to the right FEF. Furthermore, as increased ipsilateral relative to contralateral power is observed on the LPS, this points to either inhibition of the unattended field and/or disinhibition of the attended visual field.

A pronounced LDAP was observed from 540 until approximately 700 ms after cue onset (cf. Figure 3), which resembles findings from several previous studies. Nevertheless, the detailed temporal analysis suggests that the topography varies a bit over time, with initially a more occipito-parietal focus and later a more occipito-temporal focus. The source analyses suggest that this activity originates from occipital areas, which corresponds with previous results (Van der Lubbe et al., 2006), although this was not pointed out in that study. Specifically, their effects were related to the ventral intraparietal sulcus rather than occipital areas, which was biased by previous fMRI results. If we look at our LPS results in roughly the same time window with a comparable topography, then several bands appear good candidates to contribute to the LDAP. First, the θ_2_ band has the same time range and topography (cf. Figures 5, Figure 6, and Table 3). This also seems to apply to the θ_3_ band, the α_1_ and the α_2_ band (Figures 7 and 8), but less so for the β_1_ and the β_2_ bands (Figures 9 and 10). The LPS-ERP results revealed (see Table 4)that the LDAP may be explained by activities in the θ_2_ band. Most likely, the LDAP reflects the top-down influence of frontal and parietal areas on occipital areas. Furthermore, this effect again seems to concern either inhibition of the unattended visual field and/or disinhibition of the attended field.

Our ERL analyses additionally revealed a biphasic pattern with comparable occipital topographies, firstly from 700 to 800 ms, and secondly from 940 to 960 ms. The latter result may be comparable to the BRN that was observed by Grent-‘t-Jong et al. (2011), although their component occurred later and an opposite polarity was observed. Therefore, we decided to denote this component as the BRP. The differences with Grent-‘t-Jong et al. (2011) might very well be due to the longer cue-target intervals and especially the use of to-be-attended positions in the lower visual field in their experimental setup. Analyses on the LPS showed a comparable biphasic pattern in our α_1_ band, with highly similar topographies. The LPS-ERP results showed as well late occipito-parietal effects in the θ_2_, and the α_2_ bands. Grent-‘t-Jong et al. argued that the BRN reflects changes in perceptual sensitivity whereas observed effects in the α band were related to the presetting of S-R links. Here, however, both the BRP and the LPS appear to reflect the same process, and seem to concern again either inhibition of the unattended visual field and/or disinhibition of the attended field. Our findings additionally indicate that the results reported by Thut et al. (2006) and by others (referred to in our introduction) were not due to a motoric/attentional confounding or general hemispherical differences. Importantly, two recent studies provided support that these late occipital activities in the α band have a causal influence on visual perception (Dugué, Marque, & Van Rullen, 2011; Romei, Gross, & Thut, 2010). For example, Dugué et al. showed that the perception of phosphenes, which can be induced by TMS, crucially depends on the phase of EEG oscillations in the α band (see also Mathewson, Gratton, Fabiani, Beck, & Ro, 2009). This observation seems in contrast with the interpretation of the late activity in the α band by Grent-‘t-Jong et al. (2011). Interestingly, a recent magnetoencephalographic (MEG) study by Capilla, Schoffelen, Paterson, Thut, and Gross (2012) found support for different dynamics of enhancement and suppression. The ipislateral enhancement seemed to concern a transient effect and was related to the dorsal visual pathway, whereas the contralateral suppression had a more sustained influence that was related to the ventral visual pathway. The latter distinction reveals a disadvantage of the use of a double subtraction technique, as after that, the distinction between ipsilateral enhancement and contralateral suppression can no longer be made.

Some remaining LPS activity for which we found no good evoked ERL candidate is the early anterior activity in the θ_2_ band (260-460 ms). This effect might be related to the effect in the θ_3_ band visible in the LPS-ERP results, and therefore can be characterized as evoked. This activity could reflect the initial act of attentional control from anterior areas. The observed posterior activity in the β_1_ band (420-460 ms) seems too late to be incorporated by the EDAN, and too early for the LDAP, although it might reflect an earlier modulation in occipital areas that possibly has a more induced than evoked nature. Finally, intermediate (620-660 ms) anterior activity was observed in the LPS analyses in the β_1_ and β_2_ bands, which seems unrelated to the ADAN given its spectral characteristics, although this activity should be considered as evoked as comparable activity was found in our LPS-ERP results.

Given the fact that nearly all LPS activity could be explained by comparable effects on the individual ERPs, it seems that the EEG effects related to attentional orienting during the cue-target interval in general have an evoked rather than an induced nature. This extends the conclusions drawn by Makeig et al. (2002) to the current attention paradigm. Furthermore, in line with their suggestions, it may be argued that ERL features also arise from phase resetting of ongoing processes induced by the cue. Nevertheless, although probably a straightforward distinction between evoked and induced activity can be made for higher frequency bands (like the β band) this seems a bit more difficult for lower spectra especially as we used a relatively short cue-target interval of 1 s. Therefore, it seems quite relevant to verify and extend the current results with experiments employing longer cue-target intervals, which will facilitate the separation of induced and evoked activities. A related aspect worth to be mentioned is that we noticed that some effects that can be considered as evoked, as they show up after performing the LPS-ERP analyses (like the intermediate effects in the β bands), were not visible in the normal ERL analyses. As a consequence, one might argue that the variation between individuals may actually be more relevant than the intra-individual variation, at least in the current version of the Posner (1980) paradigm.

In our analyses, we observed a correlation between below threshold eye movements and the LDAP. As this correlation was only present for one time window, it seems that we safely can conclude that our observed effects are by and large due to covert orienting. Nevertheless, this observation suggests that caution is required as small below threshold saccades might induce effects. This observation also accords with ideas suggesting that there is a strong relation between the control of visual attention and the control of eye movements (e.g., see Noudoost et al., 2010; Van der Lubbe et al., 2006). Finally, we applied the Benjamini-Hochberg (1995) procedure to control the FDR (which was effective for our ERLs but not for the LPS) and the wavelet analyses on the individual ERPs. Given this outcome, we decided to apply less strict criteria (related to Talsma et al., 2001) as we had the impression that the FDR procedure is too conservative. Although we think that application of this procedure results in reliable outcomes, additional experiments seem needed to replicate and extend the currently obtained LPS results.

### Conclusions

The LPS may give us a more detailed view on the overall dynamics of visuospatial attention and a better understanding of the nature of the processes involved. On the one hand, our findings seem in line with the idea according to which visuospatial attention operates by a cascade of processes, from FEF to parietal and occipital areas. Importantly, however, this cascade of processes seems to concern inhibition of the unattended field and/or disinhibition of the attended field rather than direct gain modulation.

## Footnotes

1 In addition, authors using functional magnetic resonance imaging(fMRI) reasoned that the transient response related to cue onset in occipital cortex likely reflects processing of the cue, whereas the more sustained response after cue onset in the dorsal posterior parietal cortex (along the intraparietal sulcus [IPs]) and frontal cortex (frontal eye fields [FEF]) is thought to be related to attentional orienting (see Corbetta, Kincade, Ollinger, McAvoy, & Shulman, 2000).

2 Comparable problems have been noticed with lateralized event-related brain potentials. For example, Praamstra (2007) noticed that lateralized motor activity in a study of Wiegand and Wascher (2005) could have been confounded by lateralized activity related to visual and/or attentional processes.

3 In the studies of Grent-‘t-Jong and Woldorff (2007) and Grent-‘t-Jong, Boehler, Kenemans, and Woldorff (2011), the focus was on a later lateralized negativity, denoted as the biasing-related negativity (BRN), which may enhance perceptual sensitivity (see also Van der Stigchel, Heslenfeld, & Theeuwes, 2006). This negativity has not always been observed and seems dependent on task details like the length of the cue-target interval and the employment of relevant locations in the lower visual field.

4 In the study of Fan et al. (2007), it was observed (by comparing activity after a warning cue vs. no cue) that increased alertness was accompanied with decreased , , and power at around 200-400 ms after cue onset, whereas orienting (by comparing activity between a spatial cue vs. a warning cue) was reflected in increased power.

5 Notice that observing no effect on the ERL while observing an effect on the LPS is not sufficient to draw this conclusion, as the absence of an ERL component may be due to individual differences in the onset of specific evoked processes.
